# User-experience testing of an evidence-to-decision framework for selecting essential medicines

**DOI:** 10.1371/journal.pgph.0002723

**Published:** 2024-01-11

**Authors:** Thomas Piggott, Lorenzo Moja, Carlos A. Cuello Garcia, Elie A. Akl, Rita Banzi, Benedikt Huttner, Tamara Kredo, John N. Lavis, Holger J. Schünemann

**Affiliations:** 1 Department of Health Research Methods, Evidence, and Impact, McMaster University, Hamilton, ON, Canada; 2 Department of Family Medicine, Queens University, Kingston, ON, Canada; 3 Department of Essential Medicines and Health Products, World Health Organization, Geneva, Switzerland; 4 Department of Internal Medicine, American University of Beirut Medical Centre, Beirut, Lebanon; 5 Mario Negri Institute for Pharmacological Research IRCCS, Milan, Italy; 6 Cochrane South Africa, South African Medical Research Council, Cape Town, South Africa; 7 Department of Medicine, and Epidemiology and Biostatistics, Department of Global Health, Clinical Pharmacology, Stellenbosch University, Cape Town, South Africa; 8 McMaster Health Forum, McMaster University, Hamilton, ON, Canada; 9 Africa Centre for Evidence, University of Johannesburg, Johannesburg, South Africa; 10 Department of Biomedical Sciences, Humanitas University, Milan, Italy; 11 Department of Medicine, McMaster University, Hamilton, ON, Canada; Yale School of Medicine: Yale University School of Medicine, UNITED STATES

## Abstract

Essential medicine lists (EMLs) are important medicine prioritization tools used by the World Health Organization (WHO) EML and over 130 countries. The criteria used by WHO’s Expert Committee on the Selection and Use of Essential Medicines has parallels to the GRADE Evidence-to-Decision (EtD) frameworks. In this study, we explored the EtD frameworks and a visual abstract as adjunctive tools to strengthen the integrate evidence and improve the transparency of decisions of EML applications. We conducted user-experience testing interviews of key EML stakeholders using Morville’s honeycomb model. Interviews explored multifaceted dimensions (e.g., usability) on two EML applications for the 2021 WHO EML–long-acting insulin analogues for diabetes and immune checkpoint inhibitors for lung cancer. Using a pre-determined coding framework and thematic analysis we iteratively improved both the EtD framework and the visual abstract. We coded the transcripts of 17 interviews with 13 respondents in 103 locations of the interview texts across all dimensions of the user-experience honeycomb. Respondents felt the EtD framework and visual abstract presented complementary useful and findable adjuncts to the traditional EML application. They felt this would increase transparency and efficiency in evidence assessed by EML committees. As EtD frameworks are also used in health practice guidelines, including those by the WHO, respondents articulated that the adoption of the EtD by EML applications represents a tangible mechanism to align EMLs and guidelines, decrease duplication of work and improve coordination. Improvements were made to clarify instructions for the EtD and visual abstract, and to refine the design and content included. ‘Availability’ was added as an additional criterion for EML applications to highlight this criterion in alignment with WHO EML criteria. EtD frameworks and visual abstracts present additional important tools to communicate evidence and support decision-criteria in EML applications, which have global health impact. Access to essential medicines is important for achieving universal health coverage, and the development of essential medicine lists should be as evidence-based and trustworthy as possible.

## Introduction

Essential Medicine Lists (EMLs) are important for the prioritization and availability of medicines around the world. Essential medicine lists are a key prioritization tool to inform coverage decisions and steward limited health resources under the context of Universal Health Care [1]. The World Health Organization (WHO) Model List of Essential Medicines (MLEM) has prioritized medicines since 1977 [2]. Over 130 countries develop and use national essential medicine lists for their own context [3].

For a medicine to be deemed essential, the selection should be grounded in evidence of improved net desirable people-important health outcomes. Other dimensions than health outcomes should also be included in the evaluation process of the merits of medicines. In previous work, we found that EML committees and health practice guidelines utilize similar decision-making criteria (e.g. both consider criteria such as benefits and harms, cost and cost-effectiveness of medicines) [4]. In the context of WHO, we identified variability among health guideline topics, and opportunities to standardize the flow of medicines recommended by WHO guidelines to consideration as an essential medicine by the WHO MLEM Expert Committee. The WHO Guideline Development Handbook recommends GRADE methods to inform guideline development processes, including using Evidence-to-Decision Frameworks (EtD) [5]. These EtDs employ similar criteria to what has traditionally been requested in applications to WHO’s EML [6].

A closer link will help both EMLs and health practice guidelines to better achieve their goals of supporting evidence-based decision making [4]. Improved connection between guideline recommendations and EMLs involves synchronizing the processes used in both areas. Indeed, such coordination has sporadically been established between different WHO guideline-producing departments and the EML. In 2000, a simultaneously organized guideline and EML meeting led to a direct connection between HIV treatment guideline recommendations and essential medicine listings [4, 7]. Many WHO departments producing guidelines have mechanisms in place to assure that medicines recommended in guidelines are also assessed by the EML Expert Committee. We utilized the GRADE EtD criteria from a health guideline to support the request for addition of direct-oral anticoagulants in a WHO EML application, which was found to be a useful format [8]. A forthcoming guideline from the MSIF was conceived to directly support the application to the WHO EML and was accepted by the EML in July 2023 representing the first treatments for MS on the EML [9, 10].

This work fits within the context of broader work to coordinate decision criteria and processes between different paradigms in the ecosystem of health decision-making; EMLs and guidelines are two such paradigms [11]. In the present qualitative study, we conducted user-experience testing of a proposed EtD framework for EMLs with key stakeholders engaged in EMLs including applicants, technical staff and committee decision-makers.

## Methods

### Overview

We used the honeycomb model for user-experience presented by Morville [12]. User-experience testing presents a product for key stakeholders and observes usability, and asks directed questions about key characteristics of usability to inform improvements [12]. The honeycomb model has been previously utilized in health sciences and evidence synthesis to test usability of products [13, 14]. The dimensions centre around value of the product, with other dimensions including useful, usable, findable, credible, accessible, and desirable [12]. We conducted user-experience testing interviews of key EML stakeholders to: i) explore the perceptions about an EtD framework and visual abstract as adjunctive tools to strengthen the integration of evidence and improve the transparency of decisions regarding EML applications; and ii) ascertain how EtDs tailored to EML criteria could influence the coordination of guidelines and EMLs and influence the decision-making experience as perceived by key stakeholders.

### Research protocol, ethics review and consent

We developed a research protocol in coordination with the WHO Secretariat of the Expert Committee on the Selection and Use of Essential Medicines, to ensure strong integration of research results into global and national EML processes. The Hamilton Integrated Research Ethics Board approved this research (approval #7534). We obtained written consent from all respondents in accordance with institutional protocol (see S1 Appendix).

### Reflexivity

This research was led by researchers at the McGRADE and Michael G. DeGroote Cochrane Canada Centres and WHO Collaborating Centre for Infectious Diseases, Research Methods and Recommendations (TP, HJS) in collaboration with staff from WHO Access to Medicines and Health Products Division (LM, BH), and other experts. Authors have methodological involvement in guidelines, including as members of the GRADE working group developing the GRADE EtDs, and/or as members of essential medicine list committees. The authorship group is primarily, but not entirely, from the global north. TP brings perspectives as a cis-gendered male, white, settler public health physician in Canada. He led this work and the analysis and is trained at a graduate level in qualitative and other research methods. While the author group strived to be reflexive on position and perspective in this analysis, their perspectives provide expertise but also represent values regarding guidelines and EMLs, which may influence the perspective brought to the analysis.

### Sample applications for user-experience input

**Fig 1:**
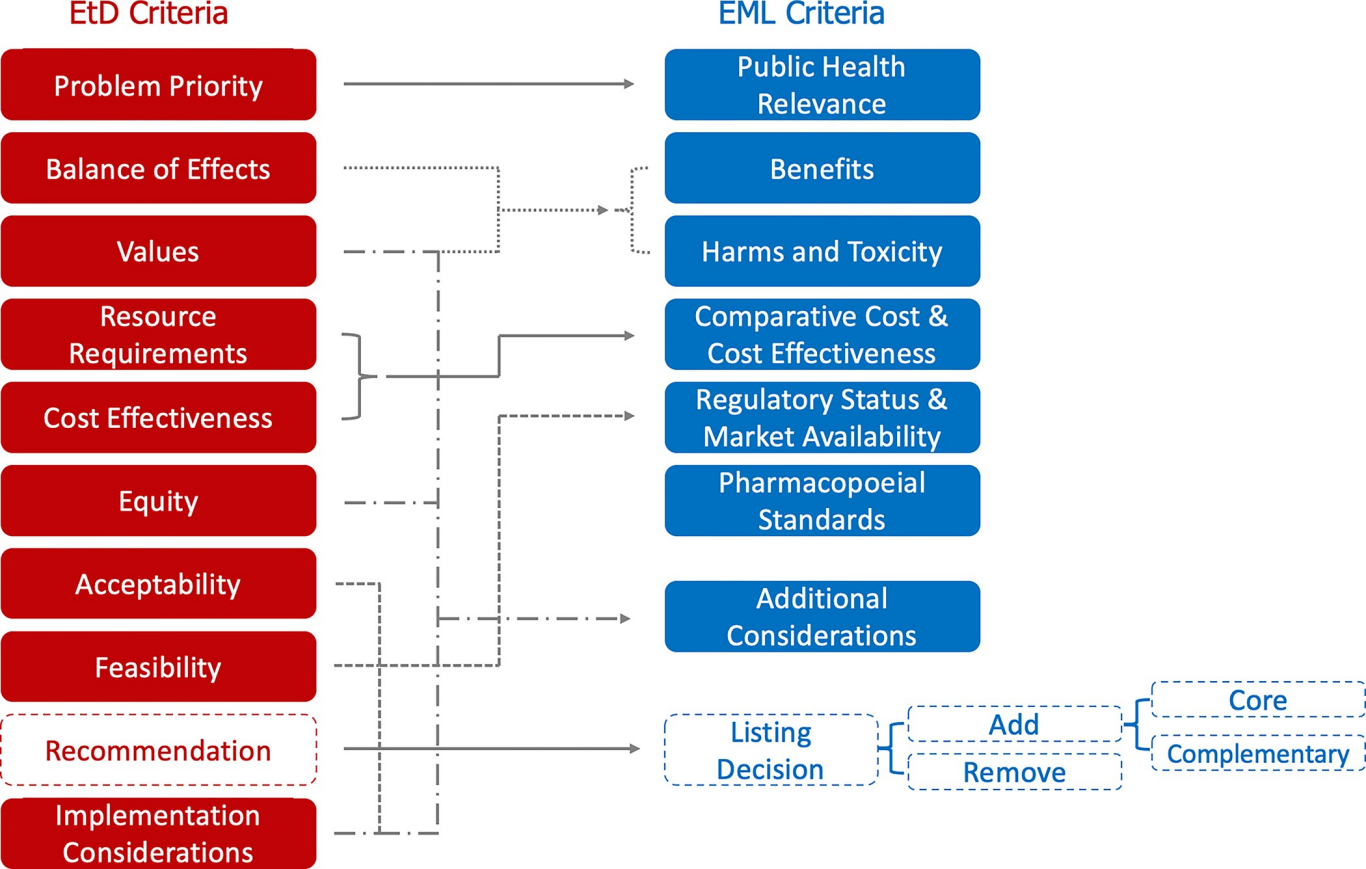
Decision criteria for guideline EtDs and mapping onto EML decision criteria [4]. This figure visualizes the decision criteria for guideline evidence-to-decision processes and EML applications. Solid lines draw connections between EtD criteria and EML criteria. Dashed lines highlight decision criteria, for a guideline this is a recommendation (strong or conditional), for an EML this is a listing decision. Listing decisions can be to add or remove a medicine from the EML.

We selected two real EML applications from the 2021 Expert Committee meeting based on representativeness of the medicines and important topics in consultation with the WHO Secretariat of the Expert Committee. The applications focused on long-acting insulin analogues (e.g. glargine) for diabetes and immune checkpoint inhibitors (e.g. pembrolizumab) for non-small cell lung cancer (NSLC) [15]. Both applications were typical applications to add medicines into the EML, addressing the merits of the medicine in long text form and in that they were not directly linked to guidelines and had not used an EtD framework (S2, S3 Appendices). To test usability of the EtD framework, we created 8-page summary EtD frameworks to capture content related to the decision criteria. The decision criteria included in the EtD were those from the traditional GRADE EtD for interventions [16, 17]. The decision criteria linking the guideline EtD criteria and EML application are presented in Fig 1 from previous work [4].

For each EML application we completed searches between May 22–30 2021 for existing systematic reviews on each decision criteria utilizing the Living Overview of Evidence (L^.^OVE) platform (Epistemonikos Foundation, Santiago, Chile). We integrated results into the EtD framework table to support judgements on EtD criteria (TP). In a guideline these judgements across EtD criteria would traditionally be made by consensus of the guideline panel, however, no panel was struck for the sample application we prepared, so based on the evidence, judgements were proposed by one researcher (TP) and subsequently reviewed by two other team members (HJS, LM). No changes to judgements on the EtD criteria (e.g. desirable, undesirable effects, equity etc.) were suggested at review. To visually summarize content and EtD criteria we also developed 1-page summary visual abstract of the EtD frameworks with input from a visualization expert (CC).

### Respondent recruitment

Our target population included both users of EML applications (committee members) and individuals preparing applications to EMLs (applicants). In collaboration with the WHO Secretariat of the Expert Committee staff we developed an initial list of stakeholders comprised of both current and former committee members and current and former applicants. We developed the list and invited individuals with attention to diversity across geographic, gender, racial, organization-type, and professional backgrounds to ensure equitable stakeholder input. We piloted the user-experience interviews (see below) with three committee members prior to the 2021 MLEM Expert Committee meeting to ensure their input on the application materials was not ‘contaminated’ by discussions at the 2021 MLEM Expert Committee meeting. We followed up with these three members following the 2021 MLEM Expert Committee meeting to assess how their perspectives had changed experiencing these applications. Subsequently we sent invitations via e-mail (with up to 2 follow-up invitations if no response) to complete user-experience interviews using our initial list of respondents and identifying additional respondents through respondent-driven sampling. We continued interviews until reaching consensus on data saturation across user-experience honeycomb dimensions.

### User-experience testing interviews

We recruited respondents for a video conferencing meeting via Zoom (Zoom Video Communications, California, USA). Prior to the meeting we shared six documents for the respondents to review. They included traditional EML application, EtD framework, and 1-page visualization of EtD framework for two 2021 WHO MLEM applications: checkpoint inhibitors for lung cancer, and long-acting insulin analogues for diabetes. We began interviews with screen-sharing and observation of interviewee review and interaction with the supplied documents. We then asked questions from a pre-developed semi-structured interview guide (available in S1 Appendix). The interview guide questions followed inquiry into Morville’s dimensions of usability: valuable, useful, usable, findable, credible, accessible, and desirable [12].

### User-experience qualitative data analysis

The primary interviewer (TP) engaged in journaling to support reflexive analysis after each interview and reviewed the information with the authorship group at several stages through the interview recruitment process. We audio recorded and transcribed them verbatim. Furthermore, we returned to interviewees if any clarification was required at the time of analysis. We then deidentified transcripts and uploaded them to NVIVO (v2022, QSR International, Melbourne, Australia) for qualitative data analysis which included review and coding by two interviewers according to Morville’s dimensions [12]. Finally, coded and classified quotes were thematically analysed using a deductive approach centred on Morville’s usability honeycomb and to opportunities to improve the proposed Evidence-to-Decision and visual abstract. We also coded quotes referring to feedback to improve either the EtD or visual abstract.

### Refinement of etd and visual abstract for emls based on User-Experience Analysis

The EtD was adapted from existing work of the GRADE working group for various health decision-making topics [16, 17]. The EtD for EML applications was iteratively refined in sequential meetings by a core project group (TP, LM, HJS, BH), based on interviews, with adaptations included in subsequent interviews, until satisfied that all themes around improving usability were adequately addressed. Thematic saturation was assessed considering differences in participant gender, perspective and region of work to ensure no variance in feedback was still emerging that could be attributable to these characteristics. This was reviewed and discussed through progress of the analysis with the full authorship group.

## Results

We identified and invited 18 potential users to participate: 13 individuals participated (response rate of 72%), 4 of whom we interviewed in a second follow-up interview, for a total of 17 interviews. Interviews ranged in duration between 41:35 and 52:57 minutes. Respondent characteristics are included in Table 1. We report our methods according to COREQ [18] (see S4 Appendix).

**Table 1:** User-experience interview respondent characteristics (see also S5 Appendix).

Characteristic	Characteristic	Number	Percentage
Gender	Female	5	38%
	Male	8	62%
	Other/Not Reported	0	0%
Perspective	WHO MLEM Member	2	15%
	WHO Staff	2	15%
	National EML Member	2	15%
	MLEM Applicant	7	54%
WHO Region of Work	AFRO	1	8%
	EMRO	1	8%
	EURO	4	33%
	PAHO	6	50%
	SEARO	1	8%
	WPRO	0	0%

### Coding

Coding using the pre-established user-experience honeycomb model yielded 103 locations of coding across all interview texts, with variability in coding volume from 1 to 23 instances across all interviews and a median of 7 codes per interview and 8.5 codes per coding category.

### User-experience qualitative data analysis

**Fig 2:**
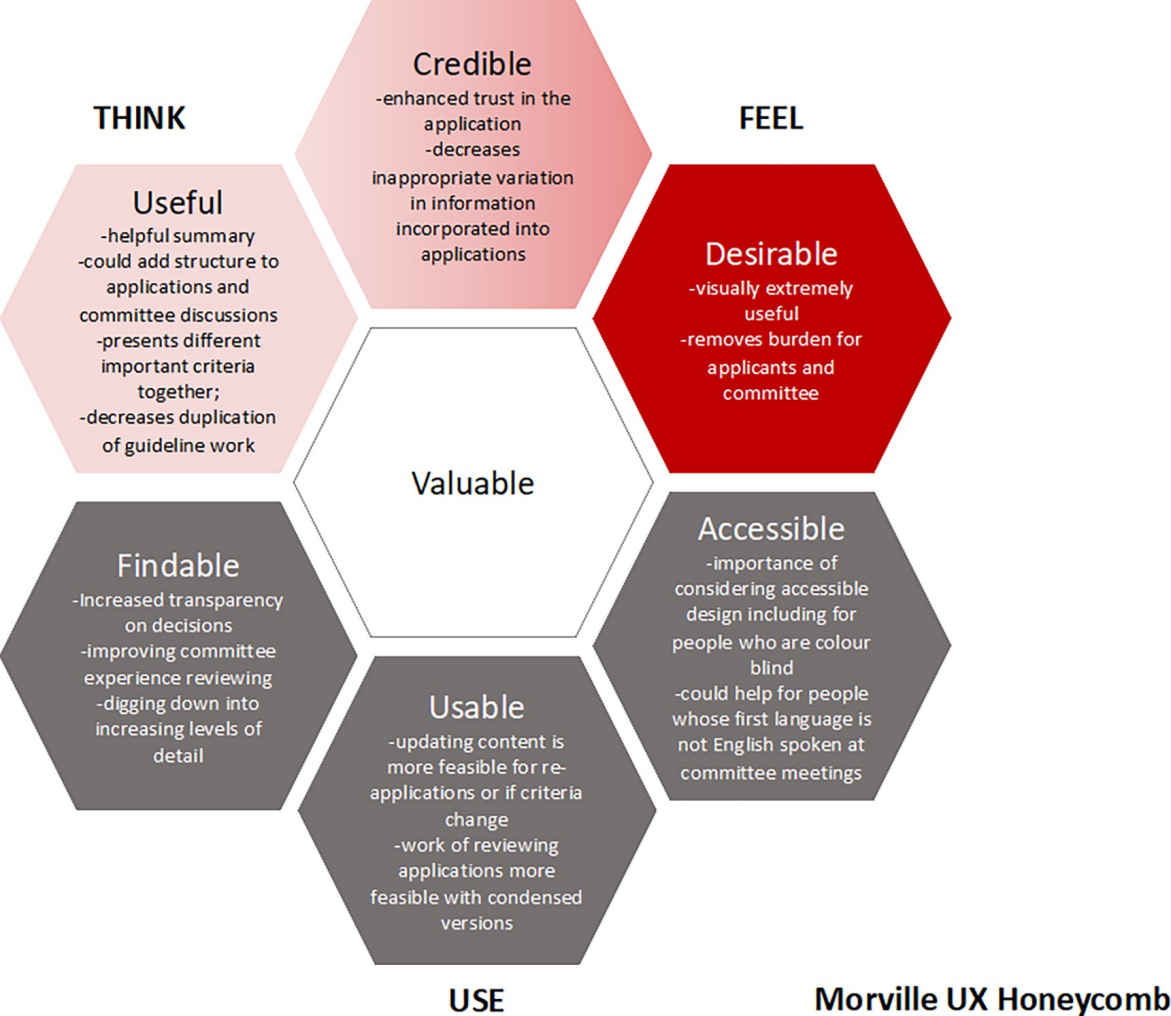
Key findings grouped by morville honeycomb model of user experience. This figure shows dimensions of the Morville user-experience honeycomb model and key themes identified under each through thematic analysis. The grey are the ‘use’ dimensions, light red are the ‘think’ dimensions and dark red ‘feel’ dimensions in the user-experience honeycomb model. Notably this includes that the EtD framework and visual abstract were found to be visually useful, more credibly incorporate evidence and useful for expert committee reviews and discussions.

The user experience of respondents yielded findings across all dimensions of Morville’s user-experience honeycomb. Key quotes supporting feedback across each dimension are included in S6 Appendix. User experience findings are also visually summarized in the honeycomb model in Fig 2. New feedback on the EtD and user experience in the honeycomb was assessed as providing no new dimensions, including by participant characteristic at the end of recruitment when the decision was made that thematic saturation had been reached.

### Summary of the information

Respondents found the products created to complement the traditional EML application, the EtD framework and visual abstract, valuable and several were emphatic on the added value. They felt given the burden of applications (nearly 90 to the WHO MLEM in 2021), it is important to have tools that can summarize the diversity of and quantity of evidence. Respondents generally felt all three products, visual abstract, EtD framework and full application should be consistent with one another and would serve different purposes depending on the level of detailed desired. Committee members assigned to reviewing and presenting the applications may make use of the full application with all its details, however other committee members, health care providers, and the general public may prefer abbreviated versions and only refer to the detailed information if they needed the specific details contained therein.

Overall, respondents felt greater methodological rigour is needed for EML applications. Respondents found the products added clarity and transparency. They also felt transparency may support implementation at the health system and health care provider level. Respondents articulated that established reporting checklists should be used as appropriate, e.g. PRISMA checklist for systematic reviews that inform applications. Further, incorporation of perspective, in particular patient/public who are impacted by EML applications was highlighted as an important suggestion. They mentioned that if applications are linked closely to existing systematic reviews or guidelines created for other purposes, they might reduce duplication of work and improve consistency of evidence synthesis across products. Finally, attention to accessibility of products was noted as important including for those who are colour-blind or whose first language is not English (as this was the language of preparation of materials).

### Content of the EtD framework for EML applications

On the basis of feedback from participants we made changes to the EtD criteria. The decision-criteria in for our proposed EtD for EMLs blends criteria in the original GRADE EtD and the decision-criteria used by the WHO EML. We changed problem priority to public health relevance, labelled desirable/undesirable effects as benefits/harms and toxicity, and added a separate criterion for availability. Values was reviewed and in contrast to other GRADE EtDs, we have brought this criteria earlier in the EtD process, because its judgement should inform the judgements on benefits and harms/toxicity. The decision proposed is: Should this medicine be on the EML: yes, list the medicine; no, do not list the medicine; remove the medicine (if already on the list); list the medicine under certain conditions (list the conditions, e.g. price reduction, research settings only). Conditional listing is not currently part of the WHO MLEM, but may be part of national EMLs. The criteria are presented in Table 2.

**Table 2:** EtD for EML decision-criteria, descriptions and judgements.

EtD for EML Criteria	Description	Judgements
Public Health Relevance	Is the medicine being evaluated for a condition of important public health relevance?	• No • Probably No • Probably Yes • Yes • Varies (if so, why?) • Don’t know
Values	Is there important uncertainty in how people value the main outcomes?	• Important Uncertainty • Possibly important uncertainty • Probably no important uncertainty • No important uncertainty
Benefits (desirable effects)	How substantial are the benefits?	• Trivial • Small • Moderate • Large • Varies (if so, why?) • Don’t know
Harms and toxicity (undesirable effects)	How substantial are the harms and toxicity?	• Trivial • Small • Moderate • Large • Varies (if so, why?) • Don’t know
Certainty of evidence	What is the overall certainty of the evidence of effects?	• Very Low • Low • Moderate • High • No included studies
	Does the balance of effects favour the medicine being considered an essential medicine?	• No • Probably No • Probably Yes • Yes • Varies (if so, why?) • Don’t know
Resources required (costs)	How large are the resources required (costs)	• Large costs • Moderate costs • Negligible costs and savings • Moderate savings • Large savings • Varies (if so, why?) • Don’t know
Cost effectiveness	Does the cost-effectiveness favour the medicine?	• Favours the medicine • Probably favours the medicine • Does not favour either the medicine or no medicine • Probably does not favour the medicine • Does not favour the medicine • Varies (if so, why?) • Don’t know
Equity	What would the impact of listing the medicine be on health equity?	• Reduced • Probably reduced • Probably increased • Increased • Varies (if so, why?) • Don’t know
Acceptability	Is the medicine acceptable to key stakeholders?	• No • Probably No • Probably Yes • Yes • Varies (if so, why?) • Don’t know
Feasibility	Is the medicine feasible to implement?	• No • Probably No • Probably Yes • Yes • Varies (if so, why?) • Don’t know
Availability	What is the regulatory status, market availability and on-the-ground availability/access of the medicine to patients?	• Not available in most settings • Probably not available in most settings • Probably available in most settings • Available in most settings • Varies (if so, why?) • Don’t know
Decision	Should this medicine be on the EML?	• Yes, list the medicine • No, do not list the medicine • Remove the medicine (if already on the list) • List the medicine under certain conditions (list conditions)

### Feedback and suggested changes to EtD framework

We incorporated suggested changes and improvements into the EtD framework presented in Table 2. Feedback centered on instructions on how to prepare the application in particular for the newly added ‘availability’ criteria. There was feedback around how availability of medicines should be operationalized and whether it should be a criterion for determining recommendations similar to feasibility, or an implementing consideration following the recommend. The feedback for the most part supported separation of availability into a separate domain, since it is a separate criterion assessed in the WHO EML application. They felt the framework would also be useful to support EML Expert Committee discussions and decisions through more succinct summary evidence than contained in traditional applications.

### Feedback and suggested changes to visual abstract

**Fig 3:**
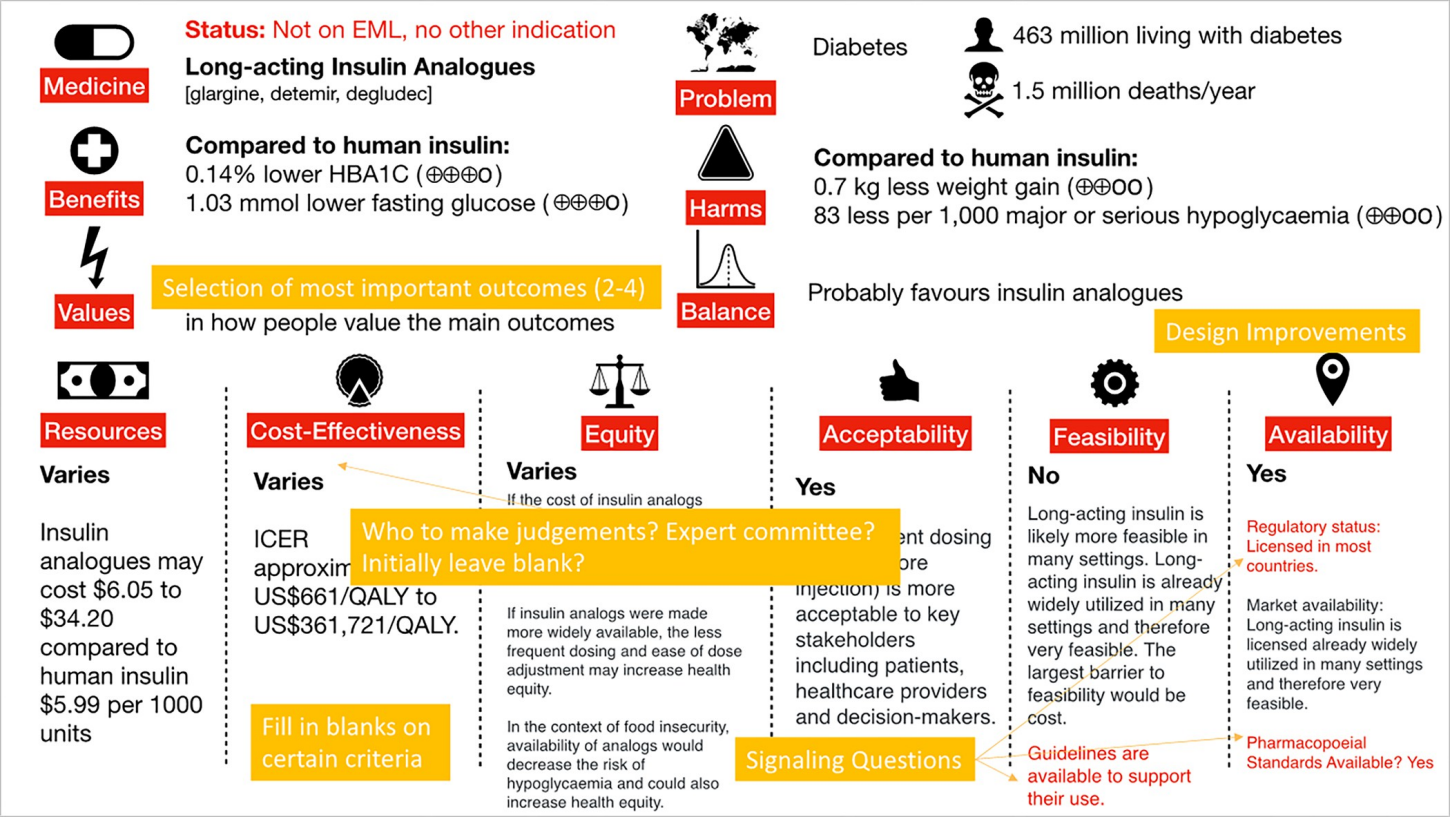
Revised visual abstract with respondent feedback displayed. The initial draft visual abstract was revised where yellow boxes show suggestions from respondents and red text shows additional text based on suggestions. “Availability” is added here as a new criterion specific for EML application.

Respondents provided constructive feedback to the visual abstract which we subsequently incorporated. Feedback is compiled visually in Fig 3. One suggestion was to add a section on current status of medicine with options including: listed, not listed, listed for other indication. Design improvements included selection of icons that were more relevant, improved readability of text, and balanced details and brevity in content provided under each domain. Respondents suggested the prompting of content for consistency and easy access to information including a “fill in the blanks” approach to criteria. They felt that the transparent communication of who was making judgements was quite important to EML processes: are these judgements for applicants to propose or EML Expert Committee members to make?

### Feedback on Instructions to EML applicants

Our interview respondents reviewed the EtD framework and input to improve these products was incorporated. A key point of feedback from applicants interviewed was around the clarity of instructions that accompany these products to applicants, and the original application. Applicants felt greater clarity in expectations and format from the WHO secretariat is important so that they could prepare the most effective application possible. Expert committee members on the other hand felt there is a balance in communicating instructions to EML applicants. They felt more directed guidance on applications may improve the quality and consistency of applications. However, they also were concerned that more instructions and rigidity could dissuade potentially valuable or important applications–submission of applications is open to any interested party including those that might not have scientific background—or create the wrong perception that significant resources or GRADE and guideline expertise are required to create an application.

For specific domains respondents felt greater clarity in instructions would be important. For example, how should cost and cost-effectiveness be considered? What settings must be reviewed or included? What methods for the estimate of cost or cost-effectiveness are appropriate. Analysis of Global North compared to Global South interviewees did not reveal any differences in feedback on the utility of the standardized process we have provided. The resources to complete an EML application were mentioned equally as a feasibility concern by interviewees from Global North and Global South. However, one respondent, who themselves was from the Global North, did note that for lower resource settings heightened standards for applications may be a greater barrier for those in the Global South.

### Feedback on Instructions to EML Committee Members for Review and Decision on Applications

Respondents emphasized the challenging task that EML Committee members have to review many applications within a relatively short timeframe and make recommendations on the upkeep of a list, at the WHO-level, now over 400 medicines long. Applications may have been previously considered by an EML committee. Documentation and clarity on previous deliberations, in addition to new evidence, is important to EML decision-making. The appointment of members, the WHO expert committee, also present a challenge and opportunity in terms of EML group process. One respondent suggested standardized training in evidence assessment would be helpful to Committee members. Respondents also suggested feedback to applicants is also important to future development of research to inform essential medicine selection. For example, if a primary barrier to listing a medicine was cost effectiveness or feasibility, feedback for what would need to change, or what additional information would be helpful is critical to improving future applications. Finally, additional instructions on how applications could be assessed in a more standardized way, e.g. the use of the AGREE tool for quality appraisal of guidelines, or development of a new tool.

## Discussion

In this qualitative study of user experience of EML applications we observed that participants perceived the tools as valuable for applicants preparing and committee members evaluating EML applications. Our results also highlight the importance of improving the coordination and connection between EMLs and guidelines, extending our previous findings that found that closely linking both processes could generate important synergy and decrease the duplication of work [4].

### Strength and limitations

As a strength, this study applies a qualitative systematic methodology, grounded in Morville’s user-experience honeycomb model, mostly used in information technology science to an important health care prioritization tool–the selection of essential medicines. We were able to work directly with decision makers and applicants to inform and improve their experience and comprehensively build upon themes that have begun to emerge from previous works [19–22]. Together, these findings should inform future methodological and group process improvements for EMLs at the global and national level.

Limitations of this study include that the majority of respondents were from the WHO EURO and PAHO regions, and our lack of success with recruiting respondents from other regions, e.g. WPRO. This may have an impact on the generalizability of the findings. However, analysis of Global North compared Global South interviewees did not reveal any differences in feedback on the utility of the standardized process we have provided and that supports that there may be little differences based on region. Further limiting generalizability of the results to national EMLs globally, only two participants with primarily national EML perspectives participated. While we sought to equitably present diverse perspectives in purposeful sampling this was not fully achieved given the participants’ characteristics and the more towards the global north oriented perspective in our author group as well as the origin of applications to the EML which more frequently are from the global north. Future work should seek to draw from these regions to obtain more perspectives and complete further testing across settings, but importantly build capacity for applications to the EML from global south countries. In terms of feasibility, the creation of visual abstracts and EtDs would place an additional work burden, particularly for nearly 90 applications, on the applicant or WHO staff, unless already produced for a guideline. This should be further assessed to determine methods of implementation, which may include prioritizing topics for applications that would most benefit by EtD/visual abstract information. However, the additional perceived burden should be worth the expected overall efficiencies through less duplication of work between guidelines and EMLs.

### Implications for practice and policy

Improved clarity for EML applicants and improved trustworthiness of the EML is a topic of significant interest to the WHO EML Secretariat and national EMLs globally [22]. The trustworthiness and criteria used to select medicines for the WHO EML has recently been subject of increased attention and critique [23]. Globally, with significant divergence of national EMLs and notable gaps from the WHO Model List of Essential Medicines, there is room to improve the methods and rigour of EML selection and the ease with which these can be adopted or adapted for national EMLs [24, 25]. Medicines on the EML should also influence guideline development recommendations so that all medicines on the EML are supported by trustworthy guidelines.

Since 2017 the MLEM is providing applicants with an application template word document. Based on a positive response to the EtD framework and visual abstracts we have created here, resources providing guidance to future applicants and supporting EML Committee members can be further expanded and updated. At the WHO level the provision of instructions and templates should be balanced with the need to maintain flexibility so that decisions may continue to be made based on the merit of medicines and not the resources available to develop the quality of the application. Nonetheless, opportunities to improve the methodological rigour and transparency in communication should be taken.

This work provides support for the connection of various paradigms of evidence synthesis for health decision-making [11, 20]. Work to develop the immune checkpoint inhibitors EtD and the visual abstract identified a Cochrane Systematic Review that addressed the same PICO question and was published the same month as the ESMO-sponsored EML application was submitted, but without an underlying systematic review. This duplication of work provides evidence for the need to better coordinate efforts to synthesize evidence for health decision-making.

### Implications for research

Our work raises additional questions on the connections between EML and other evidence-synthesis products more broadly. Further research on the feasibility, risks and benefits of aligning these processes is important. Specifically relating to the use of EtD frameworks and visual abstracts, further work should assess process to understand feasibility, desirability and benefit of this work at national levels due to our focus on the WHO Model List. Thus, it is important to address the wide variability in national EMLs, including better understanding of the processes that underlain local medicine recommendations. Greater consideration to accessibility, which were identified but inadequately explored in this work, is important across a range of barriers to accessibility including language, visual, etc. as this work continues to progress and the EtD and visual abstract are used in further settings and more detailed guidance on preparing and evaluating the various criteria evolves. Further research should explore the different national country contexts for EML globally, and whether improved usability and evidence synthesis to support EML applications, as we have communicated here, will translate to better decisions. Finally, future research should address, using evaluative or experimental methodology, the impact of the more explicit and transparent EtD process we articulate here on decisions made by EML committees.

## Conclusions

We have presented solutions to improve the methodological rigour and user-experience of applicants and Committee members for EMLs, with a focus on WHOs MLEM. We found that usability could be improved through adjunctive products such as EtD frameworks and visual abstracts. Developing these products could be developed in conjunction with linked guidelines and systematic reviews can harmonize these products, decrease duplication of work, and lead ultimately to better health decision-making and access to essential medicines.

## Supporting information

S1 Appendix Consent and semi-structured, open-ended UX interview guide. (DOCX)

S2 Appendix Evidence to decision framework “should anti-PD1 immune-checkpoint inhibitors vs. chemotherapy be used for “non-oncogene- addicted” (EGFR, ALK, and ROS1 wild type). locally advanced and metastatic non-small cell lung cancer (NSCLC)?”.
(PDF)

S3 Appendix Evidence to decision framework “should long-acting insulin analogs vs. human insulin be used for diabetes?”. (PDF)

S4 Appendix COREQ (COnsolidated criteria for REporting qualitative research) reporting checklist. (PDF)

S5 Appendix Respondent characteristics. (PDF)

S6 Appendix Coding frequency table and key quotes. (PDF)
