# Supplementary material for: User-experience testing of an evidence-to-decision framework for selecting essential medicines
Source: PLOS Glob Public Health. 2024 Jan 11;4(1):e0002723. doi: 10.1371/journal.pgph.0002723 (PMC10783770; doi:10.1371/journal.pgph.0002723)
Supplement: S1 Appendix — (DOCX) [file pgph.0002723.s001.docx]

## S1 Appendix: Consent and semi-structured, open-ended UX interview guide

**LETTER OF INFORMATION / CONSENT**

Exploring the Decision-Making Process for the Selection of Essential Medicines

Investigators:

Local Principal Investigator: PhD Student Investigator:

Dr. Holger Schünemann Dr. Thomas Piggott

Department of Health Research Methods, Department of Health Research Methods
Evidence, & Impact Evidence, & Impact

McMaster University McMaster University

1280 Main St W, HSC 2C2 1280 Main St W, HSC 2C2

Hamilton, ON, L8S 4K1 Hamilton, ON, L8S 4K1
(905) 525-9140 (905) 746-0235

E-mail: E-mail:

Purpose of the Study

You are invited to take part in this study on development of an Evidence-to-Decision making framework to support selection of Essential Medicines as an expert. The purpose of the study is to explore how an evidence to decision framework could be developed and applied to support the selection of essential medicines in the Model Essential Medicine List of the World Health Organization and National Essential Medicine Lists.

Procedures involved in the Research

The study will involve one qualitative interview lasting approximately 30-45 minutes. With your permission we hope to audio-tape the interview and later transcribe the recording. There will be several prepared questions, however, it is hoped that the interview can be more open-ended as to gain insight to your perspective on these matters. You may also be asked to use a prototype software program, while sharing your screen to test your experience and the usability of this program. The following is an example of an interview question:

- Given your knowledge and experiences with essential medicine lists, could you please begin by describing how decisions regarding the addition, maintenance, or removal of medicines from an essential medicine lists (MEML or National EML) are made?

Potential Harms, Risks or Discomforts

The risks involved in participating in this study are minimal. You may feel uncomfortable answering questions surrounding your knowledge or experiences with evidence to decision frameworks or essential medicine lists. In the event that this happens, you do not need to answer any questions that you are uncomfortable with and you can withdraw at any time during the interview. The steps taken to protect your privacy are described below.

Potential Benefits

The research will not benefit you directly. We hope to learn more about the process of selecting essential medicines. It is hoped that this will lead to the development of a framework that can be utilized by the World Health Organization and other organizations to develop essential medicine lists.

Confidentiality

You are participating in this study confidentially. Your name or any information that would allow you to be identified will be protected. No one but the researcher will know whether you participated unless you choose to tell them.

The information/data you provide will be transcribed and dissociated with your name and identity. The transcript will be kept on a password-protected computer. Once the study has been completed, the data will be destroyed.

Participation and Withdrawal

Your participation in this study is voluntary. If you decide to be part of the study, you can decide to stop (withdraw), at any time, even after signing the consent form or part-way through the study. If you decide to withdraw, there will be no consequences to you. If you wish to withdraw, please contact the research coordinator at: [piggott@mcmaster.ca](mailto:piggott@mcmaster.ca) or by phone at 905-746-0235.

Information about the Study Results

We expect to have this study completed by approximately December 2020. If you would like a brief summary of the results, please inform us how you would like them sent to you.

Questions about the Study

If you have questions or need more information about the study itself, please contact the research coordinator at: [piggott@mcmaster.ca](mailto:piggott@mcmaster.ca) or by phone at 905-746-0235.

This study has been reviewed by the Hamilton Integrated Research Ethics Board (HiREB). The HiREB is responsible for ensuring that participants are informed of the risks associated with the research, and that participants are free to decide if participation is right for them. If you have any questions about your rights as a research participant, please call The Office of the Chair of HiREB at 905.521.2100 x 42013.

CONSENT

I have read the information presented in the information letter about a study being conducted by Dr. Thomas Piggott and Dr. Holger Schünemann, of McMaster University.

I have had the opportunity to ask questions about my involvement in this study and to receive additional details I requested.

I understand that if I agree to participate in this study, I may withdraw from the study at any time. I will been given a signed copy of this form. I agree to participate in the study.

1. I agree that the interview can be audio/video recorded. Yes No

_____________________________ ________________________ _______________

Name of Participant (Printed) Signature Date

Consent form explained by:

_____________________________ ________________________ _______________

Name and Role (Printed) Signature Date

**User Testing of an Evidence-to-Decision making Framework to Support Selection of Essential Medicines**

Interview Guide

**A. Participant Information**

| **Participant no.:** |  |
| --- | --- |
| **Participant name and contact information:** | Name:  Email/Telephone: |
| **Location:** |  |
| **Date:** |  |
| **Interviewer/Note-taker:** |  |
| **Recorder interview no.:** |  |

**B. Interviewer Checklist**

***For in person interviews:***

- Confirm the date, time and location of the meeting. Send a reminder to the participant before the meeting.
- Printed copy of Interview Guide. Take notes (point-form preferred) in the spaces provided.
- Printed copy of draft list of items generated.
- Additional paper to take notes if needed.
- Audio recorder. Test the recorder before each interview.

***For interviews by telephone or online Zoom:***

- Make sure you have the land line phone number or have set up the Zoom
- For interviews by Zoom, share the meeting link with the interviewee in advance; send the PDF of the draft list of items generated.
- Confirm the date and time of the call. Send a reminder to the participant before the meeting.
- Interview Guide form to take notes. Additional paper to take notes if needed.
- Audio recorder. Test the recorder before each interview.

**C. Introduction and Consent Statement**

**Say**: Thank you very much for agreeing to participate in this interview. We are asking colleagues and other people who have been involved in the development or use of essential medicine lists their views on the current process and the potential use of a framework to assist with making medicine recommendations.

We will use your input to support this work to help support decision-making by for essential medicine lists. Our hope is that this can support the development of more rigorous and trustworthy essential medicine lists.

**Participant Consent Statement:**

**Say**: The research study has been reviewed by the Hamilton Integrated Research Ethics Board (HIREB). With your permission, the session will be recorded on tape for transcription and erased after the transcription has occurred. Transcribed data will be analyzed and coded into themes, and destroyed at the end of the study period and publication. De-identified coded themes will be destroyed after a period of 5 years. You may also withdraw your responses from the interview at any time.

Do you agree for the data collected in the study to be used anonymously in publication?

Yes  No Notes:

Do you also agree to have the interview recorded?

Yes  No Notes:

**Ask**: Do you have any questions before we proceed?

**D. Questions**

Involvement in National EML;

Duration of time involved in MLEM;

Professional background;

Cochrane/guideline involvement;

1. Before we begin I would like to just clarify your **current position** and **any involvement** or exposure you have had to **essential medicine lists**?

*Prompt: What about the WHO MLEM? Duration of time on the Expert Committee?*

**Say**: To date, we have reviewed the literature and consulted experts involved in the development of essential medicine lists. This led to the development of two draft Evidence-to-Decision frameworks on EML topics to support the decision-making process. We have shared these, as well as the two traditional applications documents, with you in advance of the meeting.

1. Comparing the Insulin Analogues application document and EtD 1: Insulin Analogues, what is your perspective on the usability for the Expert Committee in relation to the traditional application? [Share screen]

*Prompt: User-Experience Honeycomb - Is information useful? Usable? Desirable? Findable? Accessible? Credible? Valuable?*

|  |
| --- |

1. Looking at the Anti-PD1/PD-L1 antibodies application document and EtD 2: Anti-PD1/PD-L1 antibodies, what is your perspective on the usability for the Expert Committee in relation to the traditional application? [Share screen]

*Prompt* User-Experience *Honeycomb - Is information useful? Usable? Desirable? Findable? Accessible? Credible? Valuable?*

|  |
| --- |

**Additional Question Bank**

1. In what ways could using this EtD and presentation improve the consideration of the evidence by the expert committee?
2. In what ways could using this EtD and presentation hinder the consideration of the evidence by the expert committee?
3. In what ways could using this EtD and presentation affect transparency in MLEM decision-making?
4. In what ways could using this EtD and presentation affect efficiency in MLEM decision-making?
5. In what ways could using this EtD and presentation impact support national EML decision-making?
6. Should this type of presentation be used more routinely in the consideration of medicines for EMLs? Should a modified GRADEpro system be used to help?

**F. End of Interview**

**Say:** Thank you very much for your participation and feedback.
