# Supplementary material for: User-experience testing of an evidence-to-decision framework for selecting essential medicines
Source: PLOS Glob Public Health. 2024 Jan 11;4(1):e0002723. doi: 10.1371/journal.pgph.0002723 (PMC10783770; doi:10.1371/journal.pgph.0002723)
Supplement: S2 Appendix — locally advanced and metastatic non-small cell lung cancer (NSCLC)?”. (PDF) [file pgph.0002723.s002.pdf]

QUESTION

Should anti-PD1 immune-checkpoint inhibitors vs. chemotherapy be used for "non-oncogene- addicted" (EGFR, ALK, and ROS1 wild type) locally advanced and metastatic non-small cell lung cancer (NSCLC)?	
**POPULATION:**	"non-oncogene- addicted" (EGFR, ALK, and ROS1 wild type) locally advanced and metastatic non-small cell lung cancer (NSCLC)
**INTERVENTION:**	anti-PD1 immune-checkpoint inhibitors
**COMPARISON:**	chemotherapy
**MAIN OUTCOMES:**	Overall survival; Progression-free survival; Overall response rate; Adverse Events grade 3-4; Quality of Life;
**SETTING:**	
**PERSPECTIVE:**	
**BACKGROUND:**	
**CONFLICT OF INTERESTS:**	

ASSESSMENT

Problem
Is the problem a priority?

JUDGEMENT	RESEARCH EVIDENCE	ADDITIONAL CONSIDERATIONS
○ No<br>○ Probably no<br>○ Probably yes<br>● Yes<br>○ Varies<br>○ Don't know	**From Pentheroudakis MLEM Application**<br><br>Lung cancer is the most diagnosed and the first cause of death for cancer worldwide, estimating 2 million new cases and 1.7 related deaths in 2018, according to Global Cancer Observatory 2018 (5). Lung cancer is a highly lethal malignancy, with an economic impact estimated around $8 billion productivity lost in the BRICS countries (6). Moreover, in the absence of a wide coverage of an effective screening programme in place on global scale, lung cancer diagnoses occur in advanced stages (i.e. III and IV, TNM 8th) in more than 60% of cases, with highly regional variability (7-9). Lung cancer is the leading cause of cancer-related mortality in the United States and worldwide. Over 80% of the lung cancers are classified as NSCLC. Although targeted therapies have redefined the therapeutic landscape for patients with molecularly druggable NSCLC (e.g. epidermal growth factor receptor [EGFR] mutations, anaplastic lymphoma kinase [ALK] rearrangements, ROS1 rearrangements, BRAF mutations, HER2 mutations or amplifications, NTRK1-3 fusions), these therapies are ineffective in those tumours lacking such genetic alterations, the majority of NSCLC patients. However, ICI therapy has become part of the treatment of such patients, which has led to improvements in survival and quality of life. The ICI target and reactivate the immune-competent cells, i.e. T-lymphocytes and antigen-presenting cells, by inhibiting the immunosuppressive ligand PD-L1 or its receptor, PD-1, in the tumour-induced immunosuppressant milieu or by strengthening the immune-activating signals of immune-response (e.g. GITR, pro-inflammatory interleukins, interferon-gamma) (10). The approval of ICIs in NSCLC addresses an unmet need for patients considered to have a poor prognosis in advanced stage, in the absence of an indication of targeted therapy."	

Desirable Effects
How substantial are the desirable anticipated effects?

JUDGEMENT	RESEARCH EVIDENCE	ADDITIONAL CONSIDERATIONS
○ Trivial<br>○ Small<br>○ Moderate<br>● Large<br>○ Varies<br>○ Don't know	**From Dec 2020 Cochrane Review**<br>https://www.cochranelibrary.com/cdsr/doi/10.1002/14651858.CD013257.pub2/full	Evidence from original application.<br><br>Large desirable effects for expression ≥50%.

Outcomes	№ of participants (studies) Follow up	Certainty of the evidence (GRADE)	Relative effect (95% CI)	Anticipated absolute effects* (95% CI)	
				Risk with chemotherapy	Risk difference with anti-PD1 immune-checkpoint inhibitors

Overall survival	2000<br>(6 RCTs)	⊕⊕⊕◯<br>MODERATE [a]	**HR 0.68**<br>(0.60 to 0.76)	Study population	
				470 per 1,000	**119 fewer per 1,000**<br>(153 fewer to 87 fewer)
Progression-free survival	1886<br>(9 RCTs)	⊕⊕◯◯<br>LOW [a,b]	**HR 0.68**<br>(0.51 to 0.88)	Study population	
				50 per 1,000	**16 fewer per 1,000**<br>(24 fewer to 6 fewer)
Overall response rate	1672<br>(4 RCTs)	⊕⊕◯◯<br>LOW [a,b]	**RR 1.40**<br>(1.12 to 1.75)	Study population	
				287 per 1,000	**115 more per 1,000**<br>(34 more to 215 more)
Adverse Events grade 3-4	3346<br>(5 RCTs)	⊕⊕◯◯<br>LOW [a,b]	**RR 0.41**<br>(0.33 to 0.50)	Study population	
				414 per 1,000	**244 fewer per 1,000**<br>(277 fewer to 207 fewer)
Quality of Life assessed with: C30 GHS/QOL	297<br>(1 RCT)	⊕⊕◯◯<br>LOW [a,c]	**RR 1.51**<br>(1.08 to 2.10)	Study population	
				265 per 1,000	**135 more per 1,000**<br>(21 more to 292 more)

a. Downgraded one point due to risk of other bias (Carbone 2017 diKerences in baseline characteristics; Mok 2019 several protocol amendments), performance bias (Carbone 2017, Hellmann 2018, Reck 2016, Rizvi 2020, Sezer 2020), or of attrition bias (Hellmann 2018 and Rizvi 2020).
b. Downgraded one point due to inconsistency.
c. Downgraded one point due to imprecision. Results come from one single trial with relatively small sample size, or the confidence interval includes both clinically relevant values and clinically irrelevant values, thus limiting confidence to draw conclusions on an apparent lack of effect or a possible relevant effect.

Single-agent ICI
In the PD-L1 expression ≥ 50% group single-agent ICI probably improved OS compared to platinum-based chemotherapy (hazard ratio (HR) 0.68, 95% confidence interval (CI) 0.60 to 0.76, 6 RCTs, 2111 participants, moderate-certainty evidence). In this group, single-agent ICI also may improve PFS (HR: 0.68, 95% CI 0.52 to 0.88, 5 RCTs, 1886 participants, low-certainty evidence) and ORR (risk ratio (RR):1.40, 95% CI 1.12 to 1.75, 4 RCTs, 1672 participants, low-certainty evidence). HRQoL data were available for only one study including only people with PD-L1 expression ≥ 50%, which suggested that single-agent ICI may improve HRQoL at 15 weeks compared to platinum-based chemotherapy (RR: 1.51, 95% CI 1.08 to 2.10, 1 RCT, 297 participants, low-certainty evidence).
Double-agent ICI
Double-ICI treatment probably prolonged OS compared to platinum-based chemotherapy in people with PD-L1 expression ≥50% (HR: 0.72, 95% CI 0.59 to 0.89 2 RCTs, 612 participants, moderate-certainty evidence).
Trials did not report data on HRQoL, PFS and ORR according to PD-L1 groups.

Undesirable Effects
How substantial are the undesirable anticipated effects?

JUDGEMENT	RESEARCH EVIDENCE	ADDITIONAL CONSIDERATIONS
○ Large<br>● Moderate<br>○ Small	From: Dec 2020 Cochrane Review<br>https://www.cochranelibrary.com/cdsr/doi/10.1002/14651858.CD013257.pub2/full	

○ Trivial
○ Varies
○ Don't know

Outcomes	№ of participants (studies) Follow up	Certainty of the evidence (GRADE)	Relative effect (95% CI)	Anticipated absolute effects* (95% CI)	
				**Risk with chemotherapy**	**Risk difference with anti-PD1 immune-checkpoint inhibitors**
Overall survival	2000 (6 RCTs)	⨁⨁⨁◯ MODERATE [a]	**HR 0.68** (0.60 to 0.76)	Study population	
				470 per 1,000	**119 fewer per 1,000** (153 fewer to 87 fewer)
Progression-free survival	1886 (9 RCTs)	⨁⨁◯◯ LOW [a,b]	**HR 0.68** (0.51 to 0.88)	Study population	
				50 per 1,000	**16 fewer per 1,000** (24 fewer to 6 fewer)
Overall response rate	1672 (4 RCTs)	⨁⨁◯◯ LOW [a,b]	**RR 1.40** (1.12 to 1.75)	Study population	
				287 per 1,000	**115 more per 1,000** (34 more to 215 more)
Adverse Events grade 3-4	3346 (5 RCTs)	⨁⨁◯◯ LOW [a,b]	**RR 0.41** (0.33 to 0.50)	Study population	
				414 per 1,000	**244 fewer per 1,000** (277 fewer to 207 fewer)
Quality of Life assessed with: C30 GHS/QOL	297 (1 RCT)	⨁⨁◯◯ LOW [a,c]	**RR 1.51** (1.08 to 2.10)	Study population	
				265 per 1,000	**135 more per 1,000** (21 more to 292 more)

a. Downgraded one point due to risk of other bias (Carbone 2017 diKerences in baseline characteristics; Mok 2019 several protocol amendments), performance bias (Carbone 2017, Hellmann 2018, Reck 2016, Rizvi 2020, Sezer 2020), or of attrition bias (Hellmann 2018 and Rizvi 2020).
b. Downgraded one point due to inconsistency.
c. Downgraded one point due to imprecision. Results come from one single trial with relatively small sample size, or the confidence interval includes both clinically relevant values and clinically irrelevant values, thus limiting confidence to draw conclusions on an apparent lack of effect or a possible relevant effect.

Single-agent ICI
Grade 3-4 AEs may be less frequent with single-agent ICI compared to platinum-based chemotherapy (RR: 0.41, 95% CI 0.33 to 0.50, I^2 = 62%, 5 RCTs, 3346 participants, low-certainty evidence).
More information about efficacy of single-agent ICI compared to platinum-based chemotherapy according to the level of PD-L1 expression and to TMB status or specific clinical characteristics is available in the full text.
Double-agent ICI
Treatment related AEs were not reported according to PD-L1 expression levels. The frequency of grade 3-4 AEs may not differ between double-ICI treatment and platinum-based chemotherapy (RR: 0.78, 95% CI 0.55 to 1.09, I^2 = 81%, 2 RCTs, 1869 participants, low-certainty evidence).

Certainty of evidence

What is the overall certainty of the evidence of effects?

JUDGEMENT	RESEARCH EVIDENCE	ADDITIONAL CONSIDERATIONS
○ Very low<br>○ Low<br>● Moderate<br>○ High<br>○ No included studies	GRADE Certainty of Evidence Assessment from Cochrane Review 2020.	

Values

Is there important uncertainty about or variability in how much people value the main outcomes?

JUDGEMENT	RESEARCH EVIDENCE	ADDITIONAL CONSIDERATIONS
○ Important uncertainty or variability<br>○ Possibly important uncertainty or variability<br>○ Probably no important uncertainty or variability<br>● No important uncertainty or variability	A search for systematic reviews addressing values was conducted, no existing systematic reviews were identified.	A judgement on main outcomes, including survival was made that people would not have uncertainty or variability.

Balance of effects

Does the balance between desirable and undesirable effects favor the intervention or the comparison?

JUDGEMENT	RESEARCH EVIDENCE	ADDITIONAL CONSIDERATIONS
○ Favors the comparison<br>○ Probably favors the comparison<br>○ Does not favor either the intervention or the comparison<br>○ Probably favors the intervention<br>● Favors the intervention<br>○ Varies<br>○ Don't know		The judgement based on the desirable and undesirable effects, and thee certainty of effects and peoples was values was made that this favours the intervention.

Resources required

How large are the resource requirements (costs)?

JUDGEMENT	RESEARCH EVIDENCE	ADDITIONAL CONSIDERATIONS

● Large costs<br>○ Moderate costs<br>○ Negligible costs and savings<br>○ Moderate savings<br>○ Large savings<br>○ Varies<br>○ Don't know	A search for systematic reviews addressing resource requirements was conducted, one existing systematic review by de Veiga was identified comparing anti-PD1 checkpoint inhibitor to other therapies for cancers.<br><br>Direct Drug Costs: Unaffordable in a large number of countries.<br>From de Veiga 2018: "The annual cost of treating patients with NSCLC can reach as high as US$ 134,807 with nivolumab, US$ 104,244 with pembrolizumab and US$ 41,906 with docetaxel, considering only patients at the advanced stage and previously treated with platinum-based chemotherapy (Aguiar et al., 2016). To reduce this economic impact, the recommendations of economic-based scientific works involve reducing the price, the dose and the duration of treatment (Matter-Walstra et al., 2017), in addition to using predictive biomarkers when selecting patients eligible for the use of anti-PD-1s (Aguiar et al., 2016a,b,c,d, 2017a,b; Huang et al., 2016a,b; Matter-Walstra et al., 2016, 2017)."<br><br>Health System Costs: Administration may be easier and less costly.<br><br>Diagnostics: Molecular and IHC diagnosis is a vital component for the application of immunotherapy in NSCLC and involves at least PDL1 staining, EGFR and ALK analysis.	Large costs at the current pricing.<br><br>An important consideration for the listing of anti-PD1 immune checkpoint inhibitors for NSCLC as compared to melanoma, which it is currently listed for on MLEM is the incidence. Melanoma is an uncommon cancer with 2 cases per million vs 283 per million for lung cancer. NSCLC is a common form of lung cancer, however, immunotherapy target expression may not be present in the majority of NSCLC.<br><br>Therefore, the budget impact for use in lung cancer would be far greater than melanoma.

Certainty of evidence of required resources

What is the certainty of the evidence of resource requirements (costs)?

JUDGEMENT	RESEARCH EVIDENCE	ADDITIONAL CONSIDERATIONS
○ Very low<br>○ Low<br>○ Moderate<br>○ High<br>● No included studies		

Cost effectiveness

Does the cost-effectiveness of the intervention favor the intervention or the comparison?

JUDGEMENT	RESEARCH EVIDENCE	ADDITIONAL CONSIDERATIONS
● Favors the comparison<br>○ Probably favors the comparison<br>○ Does not favor either the intervention or the comparison<br>○ Probably favors the intervention<br>○ Favors the intervention<br>○ Varies<br>○ No included studies	Content original application: "A cost-effectiveness analysis has been provided for the indication of pembrolizumab frontline in advanced non-oncogene driven PD-L1 high NSCLC (22). The work aimed to measure was the incremental cost-effectiveness ratio (ICER), expressed as cost per quality-adjusted life-year (QALY) gained and the incremental cost per life-year (LY) gained. Data of safety and efficacy were derived from KEYNOTE-024 trial (23). The analysis was conducted from the perspective of a US third-party, public healthcare payer (updated to $US, year 2016 values). Pembrolizumab would be expected to result in an incremental cost of $US98,281/ QALY gained or an incremental cost of $US78,873/LY gained. Including the cost of PD-L1 testing has a very small impact on the model results. With a 5-year time horizon, the ICER was $US99,998/LY and $US122,024/QALY; with a 10-year time horizon, the ICER was $US83,065 and $US103,101/QALY. Base-case results indicated that, compared with standard of care over a 20-year time horizon, pembrolizumab would be expected to result in an additional 1.31 LYs and an additional 1.05 QALYs gained."	While these drugs have a large desirable effect, at the current price, these medicines are likely not cost-effective in most settings, particularly when diagnostic capacity is taken into account.

Equity

What would be the impact on health equity?

JUDGEMENT	RESEARCH EVIDENCE	ADDITIONAL CONSIDERATIONS

● Reduced<br>○ Probably reduced<br>○ Probably no impact<br>○ Probably increased<br>○ Increased<br><br>○ Varies<br>○ Don't know	A search for systematic reviews addressing equity was conducted, no reviews were identified.	If this drug is listed it would decrease health equity unless pricing decreases substantially.

Acceptability

Is the intervention acceptable to key stakeholders?

JUDGEMENT	RESEARCH EVIDENCE	ADDITIONAL CONSIDERATIONS
○ No<br>○ Probably no<br>● Probably yes<br>○ Yes<br><br>○ Varies<br>○ Don't know	A search for systematic reviews addressing acceptability was conducted, no reviews were identified.	These drugs are likely acceptable to patients and healthcare providers due to effectiveness and less undesirable effects than alternative regimens.<br><br>These drugs are likely not acceptable to decision-makers in most settings due to the cost.

Feasibility

Is the intervention feasible to implement?

JUDGEMENT	RESEARCH EVIDENCE	ADDITIONAL CONSIDERATIONS
● No<br>○ Probably no<br>○ Probably yes<br>○ Yes<br><br>○ Varies<br>○ Don't know	A search for systematic reviews addressing feasibility was conducted, no reviews were identified.	This intervention is feasible and already implemented in many high-income settings.<br><br>Globally this intervention is not currently feasible across most settings.<br><br>Diagnosis of gene-level expression is required before starting this treatment, and is complex and likely not feasible in many setings (e.g. outside high income countries). Molecular and IHC diagnosis is a vital component for the application of immunotherapy in NSCLC and involves at least PDL1 staining, EGFR and ALK analysis.<br><br>Challenge in time to diagnosis; in LMICs if even available, there may be a significant delay in getting PD1 expression immunohistochemistry, so it may not be available prior to the start of treatment may have to start before those results are available.<br><br>One of the largest barriers to feasibility is the current cost.<br><br>Administration of these medicines are likely easier as compared to alternative treatment regimens.

Availability

What is the regulatory status, market availability and availability of pharmacopoeial standards for this medicine?

JUDGEMENT	RESEARCH EVIDENCE	ADDITIONAL CONSIDERATIONS

○ Not Available in Most Settings<br>● Probably Not Available in Most Settings<br>○ Probably Available in Most Settings<br>○ Available in Most Settings<br>○ Varies<br>○ Don't Know	de Veiga 2018 systeematic review.	There are many settings, even high-income settings, where this treatment is not available.<br><br>Patent protection for anti-PD1 checkpoint inhibitors in ongoing in many settings, maintaining costs high and availability low.<br><br>e.g. Nivolumab: Expiring 2026 China, 2030 Brazil

SUMMARY OF JUDGEMENTS

	JUDGEMENT						
PROBLEM	No	Probably no	Probably yes	**Yes**		Varies	Don't know
DESIRABLE EFFECTS	Trivial	Small	Moderate	**Large**		Varies	Don't know
UNDESIRABLE EFFECTS	Large	**Moderate**	Small	Trivial		Varies	Don't know
CERTAINTY OF EVIDENCE	Very low	Low	**Moderate**	High			No included studies
VALUES	Important uncertainty or variability	Possibly important uncertainty or variability	Probably no important uncertainty or variability	**No important uncertainty or variability**			
BALANCE OF EFFECTS	Favors the comparison	Probably favors the comparison	Does not favor either the intervention or the comparison	Probably favors the intervention	**Favors the intervention**	Varies	Don't know
RESOURCES REQUIRED	**Large costs**	Moderate costs	Negligible costs and savings	Moderate savings	Large savings	Varies	Don't know
CERTAINTY OF EVIDENCE OF REQUIRED RESOURCES	Very low	Low	Moderate	High			**No included studies**
COST EFFECTIVENESS	**Favors the comparison**	Probably favors the comparison	Does not favor either the intervention or the comparison	Probably favors the intervention	Favors the intervention	Varies	No included studies
EQUITY	**Reduced**	Probably reduced	Probably no impact	Probably increased	Increased	Varies	Don't know
ACCEPTABILITY	No	Probably no	**Probably yes**	Yes		Varies	Don't know
FEASIBILITY	**No**	Probably no	Probably yes	Yes		Varies	Don't know
AVAILABILITY	Not Available in Most Settings	**Probably Not Available in Most Settings**	Probably Available in Most Settings	Available in Most Settings		Varies	Don't Know

TYPE OF RECOMMENDATION

Do not cover	Cover with evidence development	Cover with price negotiation	Restricted coverage	Cover
○	○	○	○	○

CONCLUSIONS

Decision

Contingent on expression ≥50%.
Contingent on laboratory ability to diagnosis PD1 expression, in a timely fashion to start treatment.

Justification

Restrictions

Implementation considerations

Monitoring and evaluation

Research priorities
