# Supplementary material for: User-experience testing of an evidence-to-decision framework for selecting essential medicines
Source: PLOS Glob Public Health. 2024 Jan 11;4(1):e0002723. doi: 10.1371/journal.pgph.0002723 (PMC10783770; doi:10.1371/journal.pgph.0002723)
Supplement: S3 Appendix — (PDF) [file pgph.0002723.s003.pdf]

QUESTION

Should long-acting insulin analogs vs. human insulin be used for diabetes?	
**POPULATION:**	diabetes
**INTERVENTION:**	long-acting insulin analogs
**COMPARISON:**	human insulin
**MAIN OUTCOMES:**	HBA1C reduction - Type 1 DM (Tricco); Fasting plasma glucose; Weight gain; Major or serious hypoglycemia;
**SETTING:**	
**PERSPECTIVE:**	
**BACKGROUND:**	
**CONFLICT OF INTERESTS:**	

ASSESSMENT

Problem
Is the problem a priority?

JUDGEMENT	RESEARCH EVIDENCE	ADDITIONAL CONSIDERATIONS
○ No<br>○ Probably no<br>○ Probably yes<br>● Yes<br>○ Varies<br>○ Don't know	**From application (Dzintars Gotham):**<br>Diabetes affected an estimated 463 million people in 2019, or 9.3% of the global population, of which 79% live in low- and middle-income countries (LMICs).(12) It was responsible for over 1.5 million deaths and 2.79% of all global disability-adjusted life years lost (DALYs) in 2019.(13) It is estimated that diabetes reduces life expectancy by 6 years when diagnosed at the age of 40.(9) Diabetes also significantly increases the risk of other non-communicable diseases, including heart disease and cancer.	

Desirable Effects
How substantial are the desirable anticipated effects?

JUDGEMENT	RESEARCH EVIDENCE	ADDITIONAL CONSIDERATIONS
○ Trivial<br>● Small<br>○ Moderate<br>○ Large<br>○ Varies<br>○ Don't know	Recent meta-analyses have found benefits for (ultra-)long-acting insulins in terms of reducing hypoglycaemic episodes and improvement in glycaemic control. The findings of these meta-analyses are more pronounced than those available at the time of earlier EML Expert Committee reviews of insulin analogues. Overall, the effect size and evidence base is arguably stronger for use in type 1 diabetes than for use in type 2 diabetes, and stronger for long-acting insulins (glargine and detemir) than for ultra-long-acting insulin (degludec).<br><br>Network Meta-analysis by Tricco et al (2021) covering 64 RCTs found that long-acting analogues led to fewer major or serious hypoglycaemic episodes (OR 0.63, 95%CI 0.51-0.79), nocturnal hypoglycaemic episodes (OR 0.74, 95%CI 0.58-0.94), reduction in HbA1c (mean difference -0.14 percentage points (95%CI -0.22 – -0.06), fasting plasma glucose reduction (mean difference -1.03 mmol/L (95%CI -1.33 – -0.73), and weight change (mean difference -0.70 kg (95%CI -1.08 - -0.32). The NMA found no significant difference for all-cause hypoglycemia, vascular complications, microvascular complications, macrovascular complications, any adverse events, serious adverse events, and drop-outs due to adverse events.<br><br>**Tricco 2021**	

Outcomes	Anticipated absolute effects* (95% CI)	Relative effect (95% CI)	№ of participants (studies)	Certainty of the evidence (GRADE)	Comments

	Risk with human insulin	Risk with long-acting insulin analogs				
HBA1C reduction - Type 1 DM (Tricco)	The mean HBA1C reduction - Type 1 DM (Tricco) was **0** %	MD **0.14 % lower** (0.22 lower to 0.06 lower)	-	9529 (25 RCTs)	⨁⨁⨁◯ MODERATE [a,b]	
Fasting plasma glucose	The mean fasting plasma glucose was **0** mmol	MD **1.03 mmol lower** (1.33 lower to 0.73 lower)	-	7928 (21 RCTs)	⨁⨁⨁◯ MODERATE [a]	
Weight gain	The mean weight gain was **0** kg	MD **0.7 kg lower** (1.08 lower to 0.32 lower)	-	7052 (15 RCTs)	⨁⨁⨁◯ MODERATE [a]	
Major or serious hypoglycemia	Study population		**OR 0.63** (0.51 to 0.79)	1294 (16 RCTs)	⨁⨁⨁◯ MODERATE [a]	
	126 per 1,000	**83 per 1,000** (69 to 102)				

a. Cochrane RoB Assessment.
b. Imprecision due to pooled confidence interval width.

Other Evidence

Type 1 diabetes
A 2018 meta-analysis covering 28 RCTs found that long-acting insulin analogues led to a reduction in general hypoglycaemia (RR 0.95, 95%CI 0.91-0.99), nocturnal hypoglycaemia episodes (RR 0.66, 95%CI 0.57–0.76) as well as a reduction in HbA1c (mean difference -0.17, 95%CI -0.23 – -0.12), and no significant difference for severe hypoglycaemia.(14)
A 2019 systematic review of severe hypoglycaemia in paediatric patients with type 1 diabetes, in real-world studies, was inconclusive as to comparison of long-acting insulin analogues to human insulin.(15)

Type 2 diabetes
A 2020 Cochrane review found significant reduction in certain measures of hypoglycaemia for insulin glargine or insulin detemir compared to insulin NPH, but no significant differences (at the p<0.05 level) in severe hypoglycaemic events, HbA1c, all-cause mortality, diabetes-related complications, or adverse events other than hypoglycaemia.

Undesirable Effects
How substantial are the undesirable anticipated effects?

JUDGEMENT	RESEARCH EVIDENCE	ADDITIONAL CONSIDERATIONS

○ Large<br>○ Moderate<br>○ Small<br>● Trivial<br>○ Varies<br>○ Don't know	(see table below)	The undesirable effects are lower with the intervention insulin analogs than comparison. Therefore undesirable effects assessed as trivial.

Outcomes	Anticipated absolute effects* (95% CI)		Relative effect (95% CI)	№ of participants (studies)	Certainty of the evidence (GRADE)	Comments
	Risk with human insulin	Risk with long-acting insulin analogs				
Weight gain	The mean weight gain was **0** kg	MD **0.7 kg lower** (1.08 lower to 0.32 lower)	-	7052 (15 RCTs)	⨁⨁⨁◯ MODERATE [a]	
Major or serious hypoglycemia	Study population		**OR 0.63** (0.51 to 0.79)	1294 (16 RCTs)	⨁⨁⨁◯ MODERATE [a]	
	126 per 1,000	**83 per 1,000** (69 to 102)				

a. Cochrane RoB Assessment.

Certainty of evidence

What is the overall certainty of the evidence of effects?

JUDGEMENT	RESEARCH EVIDENCE	ADDITIONAL CONSIDERATIONS
○ Very low<br>○ Low<br>● Moderate<br>○ High<br>○ No included studies	A certainty assessment of the evidence identified in the Tricco 2021 review was completed.	

Values

Is there important uncertainty about or variability in how much people value the main outcomes?

JUDGEMENT	RESEARCH EVIDENCE	ADDITIONAL CONSIDERATIONS
○ Important uncertainty or variability<br>○ Possibly important uncertainty or variability<br>● Probably no important uncertainty or variability<br>○ No important uncertainty or variability	A search for systematic reviews addressing values was conducted, no reviews were identified.	

Balance of effects

Does the balance between desirable and undesirable effects favor the intervention or the comparison?

JUDGEMENT	RESEARCH EVIDENCE	ADDITIONAL CONSIDERATIONS

○ Favors the comparison<br>○ Probably favors the comparison<br>○ Does not favor either the intervention or the comparison<br>● Probably favors the intervention<br>○ Favors the intervention<br><br>○ Varies<br>○ Don't know	The balance of effects probably favours the intervention, with a moderate reduction in HBA1C, FPG & large reductions in adverse events (hypoglycemia).	

Resources required

How large are the resource requirements (costs)?

JUDGEMENT	RESEARCH EVIDENCE	ADDITIONAL CONSIDERATIONS
○ Large costs<br>○ Moderate costs<br>○ Negligible costs and savings<br>○ Moderate savings<br>○ Large savings<br><br>● Varies<br>○ Don't know	For government procurement, the median price for 1000 units of analogue insulin was US$34.20 compared to US$5.99 for human insulin. When bought by patients from public sector facilities, median price for analogue insulin was $US45.03 compared to US$7.64 for human insulin. When bought by patients in the private sector, median price for analogue insulin was US$39.35 compared to US$16.65 for human insulin.<br><br>A search for systematic reviews addressing resource requirements was conducted, no review was identified.<br><br>**Documented current prices of the different insulins across countries based**<br>**Africa**<br>***Ghana***<br>Human insulin vial (10ml vials 100IU/ml) US$ 4.31<br>***Kenya***<br>Human insulin vial (10ml vials 100IU/ml) US$ 3.27 - 4.11<br>Insulin glargine vial (10ml vials 100IU/ml) US$ 11.20<br>***Nigeria***<br>Insulin glargine (per pack 5x3ml 100IU/ml pen) US$9.47 to $11.42<br>***South Africa***<br>Human insulin vial (per pack 5x3ml 100IU/ml pen) US$ 11.11<br>Insulin glargine biosimilar (per pack 5x3ml 100IU/ml cartridge; free pens) US$ 31.41<br>Insulin glargine vial originator (10ml vial 100IU/ml) US$ 36.25 per 15mls at 100IU/ml<br>**Asia**<br>***Bangladesh***<br>Insulin glargine originator (per pack 5x3ml 100IU/ml pens/cartridges) US$ 9.26 to $14.40<br>Insulin glargine biosimilar (per pack 5x3ml 100IU/ml pens/cartridges) US$ 6.05 to 12.80<br>***India***<br>Insulin glargine biosimilar [hospitals] (3ml 100IU/ml pen/cartridge) US$ 5.28<br>Insulin glargine biosimilar [pharmacy] (3ml 100IU/ml pen/cartridge) US$ 5.28 to 8.99<br>Insulin glargine originator [pharmacy] (3ml 100IU/ml pen/cartridge) US$ 9.98<br>**Europe**<br>***Bosnia and Herzigovina***<br>Human insulin (per pack 5x3ml 100IU/ml) US$ 16.4<br>Insulin glargine originator (per pack 5x3ml 100IU/ml) US$ 33.2<br>Insulin glargine biosimilar (per pack 5x3ml 100IU/ml) US$ 30.7<br>***Hungary***<br>Human insulin (per pack 5x3ml 100IU/ml) US$ 7.6<br>Insulin glargine originator (per pack 5x3ml 100IU/ml) US$ 15.2<br>Insulin glargine biosimilar (per pack 5x3ml 100IU/ml) US$ 11.6<br>***Poland***<br>Human insulin (per pack 5x3ml 100IU/ml) US$ 7.6<br>Insulin glargine originator (per pack 5x3ml 100IU/ml) US$ 8.3<br>Insulin glargine biosimilar (per pack 5x3ml 100IU/ml) US$ 8.4<br>***Scotland***<br>Human insulin (3ml 100IU/ml) US$ 7.5<br>Insulin glargine originator (3ml 100IU/ml) US$ 14.9<br>Insulin glargine biosimilar (3ml 100IU/ml) US$ 13.9	Source of current prices: Report for the 2021 WHO Expert Committee on Selection and Use of Essential Medicines on recent insulin price trends in a sample of countries (including but not necessarily limited to low- and middle-income countries), exploring key issues and suggestions for the future to enhance utilisation and funding for long-acting insulin analogues given current concerns<br><br>Prices are indicative as they are based on convinience samples.

Certainty of evidence of required resources

What is the certainty of the evidence of resource requirements (costs)?

JUDGEMENT	RESEARCH EVIDENCE	ADDITIONAL CONSIDERATIONS
○ Very low<br>○ Low<br>○ Moderate<br>○ High<br>● No included studies		

Cost effectiveness

Does the cost-effectiveness of the intervention favor the intervention or the comparison?

JUDGEMENT	RESEARCH EVIDENCE	ADDITIONAL CONSIDERATIONS
○ Favors the comparison<br>○ Probably favors the comparison<br>○ Does not favor either the intervention or the comparison<br>○ Probably favors the intervention<br>○ Favors the intervention<br>● Varies<br>○ No included studies	A search for systematic reviews addressing cost effectiveness was conducted, and identified a sysematic review published in 2017 by Shafie et al on insulin analogues in type 1 and 2 diabetes.<br><br>Of the included studies, 33 focussed on T2DM, 11 focussed on T1DM, and 6 covered both T1DM and T2DM. Twenty-one studies compared long-acting analogue insulin to insulin NPH, all of which were for high-income countries. Long-acting analogues were dominant over NPH in 5 comparisons (i.e. has both lower cost and greater benefit) and were dominated by NPH in 1 comparison (i.e. the long-acting analogue had both greater cost and lesser benefits). Apart from these cases, ICERs for long-acting insulin analogues compared to insulin NPH ranged from US$661/QALY to US$361,721/QALY.<br>Insulin analogue cost-effectiveness was strongly dependent on assumptions made regarding their benefit in reducing hypoglycaemia events and reduction in HbA1C.	The cost-effectiveness is very dependent on the cost of the insulin.

Equity

What would be the impact on health equity?

JUDGEMENT	RESEARCH EVIDENCE	ADDITIONAL CONSIDERATIONS
○ Reduced<br>○ Probably reduced<br>○ Probably no impact<br>○ Probably increased<br>○ Increased<br>● Varies<br>○ Don't know	A search for systematic reviews addressing equity was conducted, no review was identified.	If the cost of insulin analogs remains more expensive than human insulin this would probably reduce health equity.<br><br>If the availability of human insulin decreased as a result of increased use of insulin analogs, health equity would probably be reduced.<br><br>If insulin analogs were made more widely available, the less frequent dosing and ease of dose adjustment may increase health equity.<br><br>In the context of food insecurity, availability of analogs would decrease the risk of hypoglycemia and could also increase health equity.

Acceptability

Is the intervention acceptable to key stakeholders?

JUDGEMENT	RESEARCH EVIDENCE	ADDITIONAL CONSIDERATIONS

○ No<br>○ Probably no<br>○ Probably yes<br>● Yes<br>○ Varies<br>○ Don't know	A search for systematic reviews addressing acceptability was conducted, one review by Wang 2010 was identified discussing psychological resistance to insulin therapy. Frequency of injection is one contributor. Therefore less frequent injection dosing is more acceptable to patients.	Less frequent dosing (and therefore injection) is more acceptable to key stakeholders including patients, healthcare providers and decision-makers.

Feasibility
Is the intervention feasible to implement?

JUDGEMENT	RESEARCH EVIDENCE	ADDITIONAL CONSIDERATIONS
○ No<br>○ Probably no<br>● Probably yes<br>○ Yes<br>○ Varies<br>○ Don't know	A search for systematic reviews addressing feasibility was conducted, no review was identified.	Long-acting insulin is likely more feasible in many settings.<br><br>Long-acting insulin is already widely utilized in many settings and therefore very feasible. The largest barrier to feasibility would be cost.<br><br>In settings with food insecurity or in children, the incidence of hypoglycemia is likely lower with long-acting insulin analogues and therefore in these settings this would be more feasible to implement and treat diabetes.

Availability
What is the regulatory status, market availability and availability of pharmacopoeial standards for this medicine?

JUDGEMENT	RESEARCH EVIDENCE	ADDITIONAL CONSIDERATIONS
○ Not Available in Most Settings<br>○ Probably Not Available in Most Settings<br>○ Probably Available in Most Settings<br>● Available in Most Settings<br>○ Varies<br>○ Don't Know	In November 2019, the WHO Prequalification Unit published its first invitation to manufacturers for Expression of Interest (EOI) with the aim of facilitating access to biotherapeutic products, including similar biotherapeutic products (SBPs) containing the active ingredient human insulin. WHO's insulin initiative was presented at several high-level meetings. Despite the above efforts, manufacturers have yet to submit insulin dossiers to WHO Prequalification Medicines Unit.	Regulatory approval and availability present in most settings. The largest barrier to availability globally at the present time is cost.

SUMMARY OF JUDGEMENTS

	JUDGEMENT						
PROBLEM	No	Probably no	Probably yes	**Yes**		Varies	Don't know
DESIRABLE EFFECTS	Trivial	**Small**	Moderate	Large		Varies	Don't know
UNDESIRABLE EFFECTS	Large	Moderate	Small	**Trivial**		Varies	Don't know
CERTAINTY OF EVIDENCE	Very low	Low	**Moderate**	High			No included studies
VALUES	Important uncertainty or variability	Possibly important uncertainty or variability	**Probably no important uncertainty or variability**	No important uncertainty or variability			
BALANCE OF EFFECTS	Favors the comparison	Probably favors the comparison	Does not favor either the intervention or the comparison	**Probably favors the intervention**	Favors the intervention	Varies	Don't know
RESOURCES REQUIRED	Large costs	Moderate costs	Negligible costs and savings	Moderate savings	Large savings	**Varies**	Don't know

**CERTAINTY OF EVIDENCE OF REQUIRED RESOURCES**	Very low	Low	Moderate	High			**No included studies**
**COST EFFECTIVENESS**	Favors the comparison	Probably favors the comparison	Does not favor either the intervention or the comparison	Probably favors the intervention	Favors the intervention	**Varies**	No included studies
**EQUITY**	Reduced	Probably reduced	Probably no impact	Probably increased	Increased	**Varies**	Don't know
**ACCEPTABILITY**	No	Probably no	Probably yes	**Yes**		Varies	Don't know
**FEASIBILITY**	No	Probably no	**Probably yes**	Yes		Varies	Don't know
**AVAILABILITY**	Not Available in Most Settings	Probably Not Available in Most Settings	Probably Available in Most Settings	**Available in Most Settings**		Varies	Don't Know

TYPE OF RECOMMENDATION

Do not cover	Cover with evidence development	Cover with price negotiation	Restricted coverage	Cover
○	○	○	○	○

CONCLUSIONS

Decision

Justification

Restrictions

Implementation considerations

- Development of pharmacoepial standards and guidance on their use;

Monitoring and evaluation

Research priorities

- Insulin analogues in food insecure settings;
