# Supplementary material for: User-experience testing of an evidence-to-decision framework for selecting essential medicines
Source: PLOS Glob Public Health. 2024 Jan 11;4(1):e0002723. doi: 10.1371/journal.pgph.0002723 (PMC10783770; doi:10.1371/journal.pgph.0002723)
Supplement: S5 Appendix — (PDF) [file pgph.0002723.s005.pdf]

Supplementary 5 Appendix: Respondent characteristics

INTERVIEWEE	GENDER	ROLE	WHO REGION OF WORK
**1**	M	WHO Staff	EURO
**2**	M	WHO Staff	EURO
**3**	F	MLEM Expert Committee Member	EURO
**4**	M	MLEM Expert Committee Member	PAHO
**5**	M	MLEM Applicant	PAHO
**6**	F	MLEM Applicant	PAHO
**7**	M	MLEM Applicant	PAHO
**8**	F	MLEM Applicant	PAHO
**9**	M	MLEM Applicant	EURO
**10**	M	MLEM Applicant	PAHO
**11**	F	MLEM Applicant	SEARO
**12**	F	National EML Committee Member	AFRO
**13**	M	National EML Committee Member	EMRO
