# Supplementary material for: User-experience testing of an evidence-to-decision framework for selecting essential medicines
Source: PLOS Glob Public Health. 2024 Jan 11;4(1):e0002723. doi: 10.1371/journal.pgph.0002723 (PMC10783770; doi:10.1371/journal.pgph.0002723)
Supplement: S6 Appendix — (PDF) [file pgph.0002723.s006.pdf]

Supplementary 6 Appendix: Coding Frequency Table and Key Quotes

Table 1. Coding frequency table

Interviewee #	A	B	C	D	E	F	G	H	I	J	K	L	TOTAL
1	0	2	1	2	2	2	0	2	0	1	0	1	13
2	0	0	0	0	0	2	0	1	1	0	0	0	4
3	0	0	0	1	0	1	2	2	2	0	0	0	8
4	1	4	0	1	2	3	0	6	2	2	2	0	23
5	0	0	0	0	0	0	0	0	1	0	0	0	1
6	0	0	0	1	1	0	0	1	1	0	0	0	4
7	0	2	1	0	1	0	1	2	0	1	0	0	8
8	0	0	1	2	1	0	2	0	0	0	2	1	9
9	0	0	2	1	0	1	2	1	1	4	1	1	14
10	0	0	0	0	0	0	0	2	1	1	0	2	6
11	0	1	0	0	0	1	1	1	1	0	1	1	7
12	0	1	0	0	0	1	1	0	0	0	1	0	4
13	0	0	1	0	0	0	0	0	0	0	0	1	2
TOTAL	1	10	6	8	7	11	9	18	10	9	7	7	103

Table caption: A-Accessible, B-Credible, C-Desirable, D-Findable, E-Usable, F-Useful, G-visual abstract feedback, H-EtD feedback, I-traditional application feedback, J-applicant instructions, K-committee instructions, L-link to national EML.

Key Quotes

- *Findable*: EtD provides helpful summary of evidence in a more findable way to EML decision-making than longer application;

- "A high-level summary [like this visual abstract], but then being able to dig down, you find sometimes the panel is happy with high level and then sometimes they really want more detail." [Interview 1]
- "I can see, this also being really useful and I did wonder about the reviewers experience and going through all of these applications, because I think our applications are also fairly long and that's a lot of material to digest and some of the sections" [Interview 9]
- "If the goal is to make the process more transparent, having a figure like this that summarizes, people in the public can more clearly say [for example] I disagree with your judgement on equity, I don't think you're harping enough on racial bias. So I think this transparency is a very healthy process." [Interview 12]

- *Useful*: EtD could allow EML applicants to be more clear on what information the Expert Committee requires for review;
    - "So, in general I think it's a very good approach to make the different dimensions explicit right, because if you have unstructured discussion there is, maybe a tendency to focus on certain aspects and forgetting that another." [Interview 1]
    - "Well, overall, I think the presentation is really useful and it's able to summarize the key information." [Interview 3]
    - "Even on trying to answering a simple question like is this drug already in some WHO guidelines, because then different versions of the guidelines are on the website or it's difficult to find the current version and yeah I think there's room for improvement in trying to keep the different documents aligned." [Interview 5]
    - "Unfortunately, we don't have a lot of capacity to do the reviews, so here in [country] we can't afford to duplicate work and the guidelines and EML need to work together." [Interview 8]
    - "I think, having this kind of information will be very useful because there's no way that I would go read every application right, I mean in the current format." [Interview 9]
- *Desirable*: Design of the visual abstract could continue to be improved, but is pleasing;
    - "This one-pager [visual abstract] is also visually useful because you know applications are often you know very, very long and cover lot of aspects, so this is a visually extremely useful yeah." [Interview 7]
    - "Yeah, it's wonderful, I think it's really, really sharp, I like the idea of having it in a summarized systematic standardized way. It avoids sort of the descriptive

burden, not only for the [EML] committee, but also for the applicants as well" [Interview 4]

  - "the idea of having a standardized way referring to information and avoiding the sort of descriptive pieces removes the burden, not just for the committee members, but also for the applicants as well" [Interview 12]

- *Credible*: EtD could increase transparency in judgements leading to a decision to accept or reject an application, and feedback to applicants;
  - "I just wonder how much local variation there is, and then impact of this information [in the EtD and visual abstract], because obviously it depends on the disease burden [Interview 2]
  - [With traditional applications if] "you don't trust the application itself, which is again very difficult because you have to redo something like a new application by yourself. And you have to check why they included some studies and why they excluded other studies, many times, there is no description of the methodology so it's quite impossible to understand the process." [Interview 5]
  - "A systematic approach to evidence synthesis could help to increase credibility of applications and trust in evidence presented by applicant." [Interview 10]
  - "I guess the multi-layer approach is right. Then people have the option, with the audit trail, to go deeper and deeper into the details. I think the different products are useful to a range people." [Interview 12]
- *Usable*: EtD could open a process for an online software solution that would make applying and reviewing/managing applications more efficient;
  - "A digital solution would help make the process more efficient, more transparent and more evidence-based" [Interview 1]
  - "The whole application, I think is summarized here [in the visual abstract], and this was very useful, to be honest with you, they assign a few publications for each member to review in detail, you know that's okay that's when we read the entire thing, but they also asked us to review other all other applications, you know and this would be useful since there are so many other applications." [Interview 4]
  - "Well, the first time it's a lot [producing an EML application based on a guideline]. But with updates it becomes more feasible, just like guidelines" [Interview 12]

- "I think more data would be needed [for the EML committee] but then also once a decision is made, the one-pager [visual abstract] could be made available to the public and be very useful" [Interview 12]

- *Accessible*: multiple products (Full application, EtD (8 pages) and visual abstract summary) would potentially make information more accessible for Expert Committee members who at times struggle with nearly 100 applications per cycle.
    - "And we were writing … during the discussion when my English is not perfect. I have to pay attention to the different people talking during the meetings and That was my first time there and in this document was like a 700 page document that we were filing in a nutshell there, so it was something completely unmanageable so whatever idea you could have for them to streamline the process would be very, very helpful." [Interview 3]

Instructions for EML Applicants

- "So if you want to encourage application from a variety of stakeholders, probably need to find a balance between the super strict methodology, like requiring a systematic review, and then okay, a blank paper, where you can write whatever you want." [Interview 4]
- "Some domains would need like maybe some more guidance, especially you know when talking about cost, comparative cost effectiveness. We actually weren't clear what comparative cost effectiveness actually meant." [Interview 9]
- "I think there should be a requirement to you know, to base the search and synthesis of the available evidence on a systematic review with a protocol that should have at least some basic methodological requirements… to avoid this kind of stuff that we are looking at which is, you know very hard to read." [Interview 9]
- "I have unfortunately never recruited patients to be a part of an application, and that is a regret I do think that it does make an important statement to also have a patient's voice at the table as an author of an application." [Interview 10]

Instructions for EML Committee

- "Whether some basic training it's like a three-hour online module about you know guidelines systematic reviews, meta-analysis, all that, I wonder whether something like that's useful in the future for expert committee," [Interview 2]
- "the consideration of previous [decisions by Expert Committees on applications for the same medicine], even if the Group then decided to take a different decision, at least

[with standardized information and judgements in an EtD] they have the information they need to take it in a in a more informed way to jump back to the previous issue." [Interview 5]

- "The feedback that we got from [the EML Committee and secretariat] when having discussions was actually quite different from what we got in a written form. Like cost was highlighted to us in our discussions, it was not even mentioned in the in the formal written feedback." [Interview 11]

Feedback on Traditional EML Application

- "it's information overload right, and I think what our [EML committee] experts suffer from maybe you know, is a certain decision fatigue." [Interview 1]
- "I can live with the idea that the application is 40 pages, maybe if it's structured a little bit." [Interview 3]
- "it's really hard for us to go through all these applications. The search will be conducted to make sure that we're not missing any other important evidence either to support or to refute the indication so. So yes, I use either pubmed or use a Google search engine to find additional studies and in areas that I'm not more expert then I'll talk to colleagues that I know who do, you know." [Interview 4]
- "the first problem is the different format of the application so it's really difficult to navigate through some of the applications, because there are of course there's a kind of list of chapters that has to be taken, but then the content into these chapters is very different from one application to another." [Interview 3]
- "You don't trust the application itself, which is again very difficult because you have to redo something like a new application by yourself and you have to check why they included some studies and why they excluded other studies, many times, there is no description of the methodology so it's quite impossible to understand the process was false." [Interview 3]
- "The price is not a state of nature, the price is a policy variable and then, if governments are willing to issue compulsory licenses or you know not have patents on them, or something like that they can or other measures, they can change the price, and so we thought the there should be a category for something that would be called future price." [Interview 5]
- "You know, say you're a frontline professional in [sub-Sahara Africa Country] and you're interested in this process there's a lot of barriers to actually get involved and to prepare an application it's a pretty heavy lift. And so suppose there is a way to actually create like

a network, where people could actually share and collaborate on these applications I've honestly tried to do that over the past 15 years and it's tough, but I want to make sure that every voice is represented.” [Interview 10]

Feedback on EtD Framework

- “[Compared to the full application, the EtD is] still a lot, a lot of information to digest I think also probably the human mind has an inherent problem of taking into account and integrating different dimensions right.” [Interview 2]
- “Other factors were probably more important in the decision-making process than benefit and harms and those are the situations where really the discussion tends to be a little bit chaotic so having a framework to follow would probably help.” [Interview 3]
- “Difficult for for some groups, so if you want to encourage application from a variety of stakeholders, probably need to find a balance between the super strict methodology and then Okay, the blank paper, where you can write whatever you want.” [Interview 3]
- “I can see, this also being really useful and I did wonder about the reviewers experience and going through all of these applications, because I think our applications are also fairly long and that's a lot of material to digest” [Interview 8]
- “It’s useful to be very clear about the domains we deal with so in applying you can add framework to any application is very useful to be more focused, you know in distinguishing what we know about this drug”. [Interview 9]
- “I love the fact that you added a specific domain, which is also because it is a very important aspect, which is access to medicine, the availability is connected to access, which is, I guess, in line with the WHO policy it's a very important topic for the EML community, so I think this is a good addition to the classic EtD.” [Interview 9]
- “The idea of having a standardized way of referring to information and avoiding the sort of descriptive pieces removes the burden, not just for the committee members, but also for the applicants as well.” [Interview 10]

Feedback on EtD visual abstract

- “This one-pager [visual abstract] is also visually so useful because you know applications are often you know very, very long and cover a lot of aspects, so this is a visually extremely useful yeah.” [Interview 9]
- “I think the explanation is clear and obviously I understand for everything, why we made the decision the explanation is in the summary right there.” [Interview 4]

- “For this is, I didn't quite see a guideline reference here in the table. And then, it raises the question of like which guidelines are the ones that actually are would be focused on right.” [Interview 10]
- “If the goal is to make this more transparent process having a figure like this. That summarizes the criteria and people may disagree with this and say well you know I like the equity piece I don't think you're harping enough on X and you know there's a racial bias, I think those sorts of things is actually a very healthy process so that to me. Is some of the apart from the standardization that kind of making this more publicly available, and it's already it's an incredibly transparent process has is, from my point of view, in terms of posting applications and the comments and the reviews, but I’m just worried that we're not getting enough that as much as we could.” [Interview 12]
- “You have decision fatigue right, you need to make decisions in a relatively constraints timeframe, so your information needs to be accessible quickly if you need to look all kinds of different I read a lot of text it's not going to happen” [Interview 2]
